# Clinical characteristics and risk factors of enterococcal infections in Nagasaki, Japan: a retrospective study

**DOI:** 10.1186/s12879-015-1175-6

**Published:** 2015-10-16

**Authors:** Toshiki Kajihara, Shigeki Nakamura, Naoki Iwanaga, Kazuhiro Oshima, Takahiro Takazono, Taiga Miyazaki, Koichi Izumikawa, Katsunori Yanagihara, Nobuoki Kohno, Shigeru Kohno

**Affiliations:** Department of Respiratory Diseases, Nagasaki University Graduate School of Biomedical Sciences, Nagasaki, Japan; Department of Molecular Microbiology and Immunology, Nagasaki University Graduate School of Biomedical Sciences, Nagasaki, Japan; Department of Laboratory Medicine, Nagasaki University Hospital, Nagasaki, Japan; Department of Molecular and Internal Medicine, Hiroshima University, Graduate School of Biomedical Sciences, Hiroshima, Japan; Department of Chemotherapy and Mycoses, National Institute of Infectious Diseases, 1-23-1 Toyama, Shinjuku-ku, Tokyo, 162-8640 Japan

**Keywords:** Enterococcal infection, Risk factor, Retrospective study, Multivariate analysis

## Abstract

**Background:**

*Enterococcus* spp. are particularly important etiological agents of nosocomial infections. However, the clinical characteristics of and risk factors for enterococcal infections in clinical settings are poorly understood.

**Methods:**

The sample included patients with *Enterococcus* spp. infections detected from clinical samples at Nagasaki University Hospital between 2010 and 2011 and patients with enterococcal colonization (control patients). In this retrospective study, the risk factors for enterococcal infections were analyzed by comparing infected and control patients via multivariate logistic regression.

**Results:**

A total of 182 infected (mean age, 64.6 ± 18.2 years; 114 men) and 358 control patients (patients with enterococcal colonization) (mean age, 61.6 ± 22.4 years; 183 men) were included. Enterococcal infections were classified as intraperitoneal (*n* = 87), urinary tract (*n* = 28), or bloodstream (*n* = 20) infections. Cancer and hematological malignancies were the most common comorbidities in enterococcal infections. Carbapenem and vancomycin were administered to 43.8 % and 57.9 % of patients infected with *Enterococcus faecalis* and *Enterococcus faecium,* respectively. No vancomycin-resistant enterococci were isolated. Multivariate analysis identified abdominal surgery (odds ratio [OR], 2.233; 95 % confidence interval [CI], 1.529–3.261; *p ≤* 0.001), structural abnormalities of the urinary tract (OR, 2.086; 95 % CI, 1.088–4.000; *p* = 0.027), male sex (OR, 1.504; 95 % CI, 1.032–2.190; *p* = 0.033), and hypoalbuminemia (OR, 0.731; 95 % CI, 0.555–0.963; *p* = 0.026) as independent risk factors for enterococcal infections. Multivariate analysis showed abdominal surgery (OR, 2.263; 95 % CI, 1.464–3.498; *p ≤* 0.001), structural abnormalities of the urinary tract (OR, 2.634; 95 % CI, 1.194–5.362; *p* = 0.008), and hypoalbuminemia (OR, 0.668; 95 % CI, 0.490–0.911; *p* = 0.011) were independent risk factors for *E. faecalis* infection. Finally, immunosuppressive agent use (OR, 3.837; 95 % CI, 1.397–10.541; *p* = 0.009) and in situ device use (OR, 3.807; 95 % CI, 1.180–12.276; *p* = 0.025) were independent risk factors for *E. faecium* infection.

**Conclusions:**

These findings might inform early initiation of antimicrobial agents to improve clinical success.

## Background

Enterococci are gram-positive, catalase-negative, and non-spore-forming facultative anaerobic bacteria. Enterococci are generally commensal and may aid with digestion and other gut metabolic pathways [1, 2]. The two most common species responsible for enterococcal infections in humans are *Enterococcus faecalis* and *Enterococcus faecium*. Some *Enterococcus* species are used in probiotics to treat diarrhea and improve host immunity [3]. While most *Enterococcus* species are commensal organisms, *E. faecalis* and *E. faecium* have become particularly important etiological agents of nosocomial infections [4, 5]; both can survive in hospital environments and colonize patients, causing infections such as urinary tract infections, hepatobiliary sepsis, endocarditis, surgical wound infections, bacteremia, and neonatal sepsis. Previous reports have shown that pharyngeal or intestinal colonization of enterococci are risk factors for enterococcal bacteremia, which is associated with increased mortality, particularly in immunocompromised patients [6–8]. Enterococci, one of the most common nosocomial pathogens, incur a high mortality rate. The increasing use of antineoplastic, biological, and other immunosuppressive agents is one reason enterococcal infections have become a major cause of nosocomial infections [9, 10]. This study aimed to describe the epidemiology clinical characteristics of and risk factors for enterococcal infections.

## Methods

### Study design

We performed a retrospective study of patients in whom enterococci were detected in clinical samples between January 1, 2010, and December 31, 2011, at Nagasaki University Hospital, an 862-bed tertiary care and teaching hospital in Nagasaki, Japan, to identify the risk factors for enterococci infection. Medical records were reviewed for all patients with samples that were culture-positive for enterococcal organisms. A total of 571 cases positive for *Enterococcus* spp (540 inpatients, 31 outpatients) and 583 isolates were analyzed, including 12 duplicated cases (in which the enterococci were re-isolated >3 months after the first isolation, or multiple enterococci were isolated from a patient). This retrospective study, including the analysis and release of clinical data, was approved by the Ethical Committee of Nagasaki University Hospital.

### Enterococcal infection criteria

Enterococcal infections were identified on the basis of clinical symptoms (temperature > 37.5 °C and organ-specific symptoms), laboratory data (white blood cell count > 9100/mm^3^ and C-reactive protein > 0.17 mg/dL), and bacteriological tests (monomicrobial culture or the same organisms isolated from two organs). Microorganisms were considered the causative pathogens of cellulitis or cutaneous abscesses when they were cultured from abscess fluid, tissue specimens, or blood. Those who did not present with distinctive symptoms of infection but in whom enterococci had been detected were considered to be uninfected control patients (colonized patients).

### Microbiological methods

Antimicrobial resistance to ampicillin, vancomycin, linezolid, imipenem, levofloxacin, and teicoplanin was detected by measuring minimum inhibitory concentrations using the PMIC-85 test panel for gram-positive bacteria (BD Diagnosis, Sparks, MD, USA) on a BD PHOENIX microbiology system (Becton, Dickinson and Company, NJ, USA). Categorical interpretations were assigned according to Clinical Laboratory Standards Institute recommendations [11].

### Statistical analysis

Data were analyzed by Pearson’s *χ*^2^ test or Fisher’s exact test when the expected count in any category was >5, with Bonferroni post-hoc tests. Continuous non-normally distributed variables were analyzed by the Mann–Whitney *U*-test. Multivariate logistic regression analysis with forward stepwise selection was performed to estimate the adjusted odds ratios (ORs) and 95 % confidence intervals (CIs) of the variables to determine the potential risk factors for infection.

The Hosmer–Lemeshow test was performed as a measure of goodness of fit. The level of significance was set at *p* < 0.05. All statistical analyses were performed using IBM SPSS for Windows version 20.0 (IBM Corp., Armonk, NY, USA).

## Results

### Study population and patient characteristics

The epidemiological variables, infection types, antimicrobial therapies, and culture-positive specimens from 583 *Enterococcus* spp. case isolates are summarized in Table 1. These corresponded to 182 infected patients (33.7 %), including 74 (40.7 %) intra-peritoneal infections, 13 (7.1 %) intra-peritoneal infections after liver transplantation, and 29 (15.9 %) urinary tract infections; moreover, enterococci colonization was noted in 358 hospitalized patients. In intraperitoneal infections after liver transplantation, the number of *E. faecium* infections was significantly greater than that of *E. faecalis* infections (*p* < 0.001). On the other hand, in urinary tract infections, the number of *E. faecalis* infections was significantly greater than that of *E. faecium* infections (*p* = 0.016). *Enterococcus* spp. were identified from urine (*n* = 32; 17.6 %), abscess discharge (*n* = 45; 24.7 %), and blood (*n* = 37; 20.3 %). Abscess discharge was the most common *E. faecalis* culture-positive specimen type, occurring in 38 (25.7 %) infected patients. Peritoneal fluid and abscess discharge were the most common *E. faecium* culture-positive specimen types, with eight (21.1 %) infected patients.

**Table 1:** Epidemiological variables and infection types

	Total *n* = 182	*E. faecalis n* = 144	*E. faecium n* = 38
Age, mean (SD), years	64.6 (18.2)	65.0 (19.3)	64.8 (13.5)
Male sex *n*, (%)	114 (62.6)	89 (61.8)	25 (65.8)
Type of infection *n*, (%)			
Intra-peritoneal infections	74 (40.7)	59 (41.0)	15 (39.5)
Intra-peritoneal infections after liver transplantation	13 (7.1)	4 (2.8)	9 (23.7)
Urinary tract infections	29 (15.9)	29 (20.1)	0 (0.0)
Febrile neutropenia	16 (8.8)	11 (7.6)	5 (13.2)
Bone and soft-tissue infections	17 (9.3)	14 (9.7)	3 (7.9)
Blood stream infections	20 (11.0)	15 (10.4)	5 (13.2)
Pulmonary infections	10 (5.5)	10 (6.9)	0 (0.0)
Vascular grafts infections	3 (1.6)	2 (1.4)	1 (2.6)
Comorbidity *n*, (%)			
Cancer and hematologic malignancy	65 (35.7)	57 (39.6)	8 (21.1)
Hematologic malignancy	22 (12.1)	14 (9.7)	8 (21.1)
Solid organ transplantation	19 (10.4)	10 (6.9)	9 (23.7)
Bone marrow and stem cell transplantation	9 (4.9)	3 (2.1)	6 (15.8)
Diabetes mellitus	14 (7.7)	12 (8.3)	2 (5.3)
Collagen vascular disease	12 (6.6)	9 (6.3)	3 (7.9)
Chronic kidney disease	53 (29.1)	40 (27.8)	13 (34.2)
Structural abnormality of urinary tract	22 (12.1)	21 (14.6)	1 (2.6)
Hemodialysis	12 (6.6)	10 (6.9)	2 (5.3)
Antimicrobial therapy *n*, (%)			
Vancomycin	48 (26.4)	26 (18.1)	22 (57.9)
Linezolid	13 (7.1)	3 (2.1)	8 (21.1)
Teicoplanin	14 (7.7)	6 (4.2)	8 (21.1)
Meropenem	37 (20.3)	37 (25.7)	0 (0.0)
Imipenem/cilastatin	15 (8.2)	15 (10.4)	0 (0.0)
Biapenem	2 (1.1)	2 (1.4)	0 (0.0)
Doripenem	9 (4.9)	9 (6.3)	0 (0.0)
Piperacillin	10 (5.5)	10 (6.9)	0 (0.0)
Tazobactam/piperacillin	18 (9.9)	18 (12.5)	0 (0.0)
Ampicillin	2 (1.1)	2 (1.4)	0 (0.0)
Sulbactam/ampicillin	6 (3.3)	6 (4.2)	0 (0.0)
Levofloxacin	21 (11.5)	21 (14.6)	0 (0.0)
Ciprofloxacin	3 (1.6)	3 (2.1)	0 (0.0)
Tosufloxacin	1 (0.5)	1 (0.7)	0 (0.0)
Combination therapy	18 (9.9)	18 (12.5)	0 (0.0)
Culture-positive specimens *n*, (%)			
Urine	32 (17.6)	27 (18.8)	1 (2.6)
Sputum	9 (4.9)	9 (6.3)	0 (0.0)
Blood	37 (20.3)	25 (17.4)	6 (15.8)
Peritoneal fluid	32(17.6)	24 (16.7)	8 (21.1)
Pleural fluid	1 (0.5)	1 (0.7)	0 (0.0)
Bile	16 (8.8)	10 (6.9)	6 (15.8)
Discharge from abscess	45(24.7)	37 (25.7)	8 (21.1)
Central venous catheter tip	4 (2.2)	4 (2.8)	0 (0.0)
Throat swab	5 (2.7)	4 (2.8)	1 (2.6)
Vascular graft	3 (1.6)	2 (1.4)	1 (2.6)
Polymicrobial culture	83 (45.6)	70 (48.6)	13 (34.2)

The overall mean ± SD age of the patients was 64.6 ± 18.2 years, and 114 patients (62.8 %) were men. Enterococcal infections were most frequently found in patients with cancer and hematologic malignancy (65 patients; 35.7 %), structural abnormalities of the urinary tract (22 patients; 12.1 %), and solid organ transplant (19 patients; 10.4 %). Cancer and hematologic malignancies (39.6 %) and structural abnormalities (14.6 %) were most common in patients with *E. faecalis* infections. Meanwhile, solid organ transplantation (23.7 %) and hematologic malignancy (21.1 %) were most common in patients with *E. faecium* infections. Compared with *E. faecalis*, bone marrow and stem cell transplantation were significantly common in *E. faecium* infections (*p* = 0.027).

Meropenem was the most commonly prescribed antibiotic for *E. faecalis* (*n* = 37, 25.7 %). Among the patients infected with *E. faecalis*, 18 (12.5 %) received combination therapies consisting of anti-MRSA(Methicillin-resistant *Staphylococcus aureus*) agents and other antibiotics. Vancomycin was the most commonly prescribed antibiotic for *E. faecium* infections (*n* = 22, 57.9 %).

### Antimicrobial susceptibilities of 583 enterococcal strains

Antimicrobial susceptibility was determined for the 583 isolates. Amoxicillin resistance was observed in 1.1 % of *E. faecalis* and 90.8 % of *E. faecium* isolates. In this study, no vancomycin-resistant enterococci were isolated, identical to results of a previous Japanese report [12]. Imipenem resistance was found in 0.9 % of *E. faecalis* and 92.4 % of *E. faecium* isolates. Levofloxacin resistance was found in 19.9 % of *E. faecalis* and 96.9 % of *E. faecium* isolates. Only one *E. faecium* isolate had teicoplanin resistance. There were no linezolid-resistant enterococci among the 550 enterococcal isolates.

### Risk factors for *Enterococcus* spp. infection

A total of 182 infected patients and 358 colonized control patients were compared to determine the risk factors for *Enterococcus* spp. infection (Table 2). In multivariate analysis, abdominal surgery was the strongest risk factor for enterococcal infection (adjusted OR, 2.233; 95 % CI, 1.529–3.261; *p ≤* 0.001). Other significant risk factors for infection were structural abnormalities of the urinary tract (adjusted OR, 2.086; 95 % CI, 1.088–4.000; *p* = 0.027), male sex (adjusted OR, 1.504; 95 % CI, 1.032–2.190; *p* = 0.033), and hypoalbuminemia (adjusted OR, 0.731; 95 % CI, 0.555–0.963; *p* = 0.026).

**Table 2:** Risk factors for enterococcal infection

	Enterococcal infections					*Enterococcus faecalis* infections					*Enterococcus faecium* infections				
	Infections	Controls	Univariate	Multivariate		Infections	Controls	Univariate	Multivariate		Infections	Controls	Univariate	Multivariate	
	*n* = 182	*n* = 358	*p* value	Adjusted OR (95 % CI)	*p* value	*n* = 144	*n* = 275	*p* value	Adjusted OR (95 % CI)	*p* value	*n* = 38	*n* = 83	*p* value	Adjusted OR (95 % CI)	*p* value
Age, years, mean (SD)	64.6 (18.2)	61.6(22.4)	0.458	-	-	65.0 (19.3)	60.7 (23.0)	0.187	-	-	64.8(13.5)	64.7 (20.1)	0.351	-	-
Sex (male), *n* (%)	114 (62.6)	183 (51.1)	0.011	1.504 (1.032-2.190)	0.033	89 (61.8)	142 (51.6)	0.047	-	-	25 (65.8)	41 (49.4)	0.094	-	-
Comorbidities, *n* (%)															
Diabetes mellitus	38 (20.9)	80 (22.3)	0.697	-	-	31 (21.5)	63 (22.9)	0.748	-	-	7 (18.4)	17 (20.5)	0.793	-	-
Cancer and hematologic malignancy	96 (52.7)	171 (47.8)	0.274	-	-	77 (53.5)	131 (47.6)	0.257	-	-	19 (50.0)	40 (48.2)	0.854	-	-
Chronic kidney disease	52 (28.6)	109 (30.4)	0.653	-	-	39 (27.0)	84 (30.5)	0.46	-	-	13 (34.2)	25 (30.1)	0.654	-	-
Structural abnormality of the urinary tract	21 (11.5)	22 (6.1)	0.029	2.086 (1.088–4.000)	0.027	20 (13.8)	17 (6.2)	0.008	2.634 (1.294–5.362)	0.008	1 (2.6)	5 (6.0)	0.427	-	-
Hemodialysis	12 (6.6)	33 (9.2)	0.297	-	-	10 (6.9)	20 (7.3)	0.902	-	-	2 (5.3)	13 (15.6)	0.109	-	-
Therapy for comorbidities, *n* (%)															
Abdominal surgery	90 (49.5)	102 (28.5)	0	2.233 (1.529–3.261)	0	68 (47.2)	72 (26.2)	0	2.263 (1.464–3.498)	0	22 (57.9)	30 (36.1)	0.025	-	-
Steroids	42 (23.1)	75 (20.9)	0.571	-	-	23 (16.0)	47 (17.1)	0.771	-	-	19 (50.0)	28 (33.7)	0.09	-	-
Immunosuppressive agents	30 (16.5)	41 (11.5)	0.102	-	-	17 (11.8)	29 (10.5)	0.696	-	-	13 (34.2)	12 (14.5)	0.013	3.837 (1.397–10.541)	0.009
Chemotherapy	30 (16.5)	62 (17.3)	0.807	-	-	23 (16.0)	47 (17.1)	0.771	-	-	7 (18.4)	15 (18.1)	0.963	-	-
Urinary catheterization, *n* (%)	75 (41.2)	145 (40.5)	0.875	-	-	57 (39.6)	103 (37.5)	0.671	-	-	18 (47.4)	42 (50.6)	0.742	-	-
Using in situ device, *n* (%)	120 (65.9)	190 (53.1)	0.004	-	-	86 (59.7)	132 (48.0)	0.023	-	-	34 (89.5)	58 (69.9)	0.02	3.807 (1.180-12.276)	0.025
Hemoglobin (g/dL), mean (SD)	9.89 (1.97)	10.2 (2.0)	0.093	-	-	10.0 (2.0)	10.4 (2.1)	0.17	-	-	9.37 (1.96)	9.63 (1.48)	0.143	-	-
Albumin (g/dL), mean (SD)	2.86 (0.63)	3.06 (0.87)	0.01	0.731 (0.555-0.963)	0.026	2.86 (0.63)	3.13 (0.93)	0.002	0.668 (0.490-0.911)	0.011	2.88 (0.63)	2.85 (0.55)	0.823	-	-
In-hospital mortality (%)	8.2	8.9	0.786	-	-	9	7.3	0.527	-	-	5.3	14.5	0.144	-	-
Total hospital stay (days), mean (±SD)	80.9 (95.0)	74.0 (110)	0.053	-	-	77.5 (101)	70.8 (95.0)	0.223	-	-	93.8 (68.3)	86.8 (149)	0.04	-	-
	Hosmer–Lemeshow: *p* = 0.571					Hosmer–Lemeshow: *p* = 0.825.					Hosmer–Lemeshow: *p* = 0.677.				

### Risk factors for *E. faecalis* infection

A total of 144 infected and 275 control patients were compared to determine the risk factors for *E. faecalis* infection (Table 2). Structural abnormalities of the urinary tract conferred the highest risk of *E. faecalis* infection (adjusted OR, 2.634; 95 % CI, 1.294–5.362; *p* = 0.008) on multivariate analysis. Other significant risk factors were abdominal surgery (adjusted OR, 2.263; 95 % CI, 1.464–3.498; *p ≤* 0.001) and hypoalbuminemia (adjusted OR, 0.668; 95 % CI, 0.490–0.911; *p* = 0.011).

### Risk factors for *E. faecium* infection

A total of 38 infected patients and 83 control patients were compared to determine the risk factors for *E. faecium* infection (Table 2). Immunosuppressive agent use (adjusted OR, 3.837; 95 % CI, 1.397–10.541; *p* = 0.017) and in situ device use (OR, 3.807; 95 % CI, 1.180–12.276; *p* = 0.025) were significant risk factors for *E. faecium* infection.

## Discussion

This retrospective study analyzed the epidemiology, characteristics, and risk factors for vancomycin-susceptible enterococcal (VSE) infections compared with colonized control patients on the basis of medical records.

In the United States, enterococci are recognized as important causative pathogens of catheter-related bloodstream infections, urinary tract infections, and cellulitis [2, 9, 10].

The independent risk factors for vancomycin-resistant enterococcal (VRE) infection were reported previously. Kim et al. compared the VRE “infected” and “colonized” groups and reported combined infection with bacteria other than VRE, presence of a hemodialysis catheter, and duration of vancomycin use were the independent risk factors for VRE infection [13]. Olivgeris et al. reported that cortisone use, third- or fourth-generation cephalosporins, enteral nutrition, and VRE colonization were the risk factors for developing enterococcal infection in critical ill patients [14]. Zaas et al. reported that the use of vancomycin, gastrointestinal procedures, diabetes mellitus, and acute renal failure could be risk factors for enterococcal bloodstream infections in malignant patients who colonized VRE [15]. As shown in these reports, the risk factors of enterococcal infections, especially VRE, are variable depending on patient background. In addition, the risk factors for VSE infections, which are highly prevalent in Japan, remain unknown. In our study, structural abnormalities of the urinary tract, abdominal surgery, immunosuppressive agent use, male sex, hypoalbuminemia, and the use of in situ devices are the risk factors of VSE infections.

Moreover, Sugiura et al. reported that enterococci are the most common causative pathogens of intra-abdominal infections after pancreaticoduodenectomy [16] and recommend accounting for enterococcal infections when selecting antimicrobials for empirical treatment for intra-abdominal infections after abdominal surgery. Furthermore, Kim et al. report that liver transplantations are an independent risk factor for enterococci intra-abdominal infections [17]. A high incidence of enterococcal infection was also found in the current study, and 17 of the 42 liver transplantations performed during 2010–2011 at our hospital were complicated by enterococcal infections (which corresponds to a high complication incidence of 42 %). In addition, according to an analysis of isolates from intra-abdominal infections after liver transplantations, the isolation frequency of *E. faecium* (23.7 %) is higher than that of *E. faecalis* (2.8 %) (*p* < 0.001). Enterococci, particularly *E. faecium*, are considered important causative pathogens of infections in liver transplantation recipients. In cases in which healthcare-associated infections are suspected after liver transplantation, the intra-abdominal infection complication guidelines of the 2010 Surgical Infection Society and Infectious Diseases Society of America recommend selecting antimicrobial agents that cover enterococci (e.g., ampicillin, piperacillin/tazobactam, vancomycin) to treat infections in patients administered antibiotics that do not cover enterococci, such as cephalosporin; in immunocompromised patients; or patients with valvular heart disease, prosthetic heart valves, or artificial blood vessels [18]. In addition, the resistance to anti-MRSA drugs, especially the increasing prevalence of VRE present in nosocomial infections, has become a major problem in the United States and Europe [5]. Since VRE was first reported as a VanA type-resistant *E. faecium* in 1988 in the United Kingdom and France, and VanB type-resistant *E. faecalis* in 1989 [19, 20], its prevalence has been increasing annually; reports from the United States indicate that 62.3–82.6 % of *E. faecium* isolates detected in nosocomial infections are VRE [9]. In Europe, it is most prevalent in Ireland (44.0 %), followed by Portugal (23.3 %) and Greece (17.1 %) [5, 21]. In China, the prevalence of vancomycin-resistant *E. faecium* is 2.7 % while that of vancomycin-resistant *E. faecalis* is 6.5 % [22]; the detection rates in Asia, including Japan, are low [12, 23], and these species were not isolated in the present study.

Interestingly, *E. faecalis* was identified as the causative pathogen in all cases of enterococcal urinary tract infections in the present study. Enterococci are causative pathogens in complicated urinary tract infections and feature a high isolation frequency [24]. Urine from children with congenital urinary tract disorders shows a high frequency of *E. faecalis* isolation [25]; in the present study as well, nearly all cases involving *E. faecalis* isolated from the urine were accompanied by congenital and acquired urinary tract disorders. Moreover, the frequency of *E. faecalis* isolation in bacterial cultures from ureteral stents is reportedly high [26]; likewise, in the present study, cases with acquired urinary tract disorders and *E. faecalis* isolation coincided with the presence of artificial devices from procedures such as nephrostomies and stent placements. Many factors related to the onset of urinary tract infection by *E. faecalis* have been reported. For example, enterococcal polysaccharide antigen is reportedly involved in the onset of ascending urinary tract infections since it binds to epithelial cells followed by biofilm formation and/or resistance to phagocytosis by polymorphonuclear leukocytes [27]. On the other hand, fewer reports have examined the pathogenicity of *E. faecium* affecting the onset of urinary tract infection than those on *E. faecalis* [10], which may be related to the results of our present clinical study. Structural abnormalities of the urinary tract might be unique compared with the risk factors of VRE infections, mainly caused by *E.faecium*, indicating that we should closely monitor patients with primary or secondary urinary tract abnormalities, stent placement, and nephrostomy for VSE infections, especially *E. faecalis*.

The use of immunosuppressive agents was an independent risk factor for *E. faecium* infection. Unlike *E. faecalis*, *E. faecium* recognizes peptidoglycan differences and has different acid production mechanisms that result in growth rate differences [2]. The recently increasing incidences of collagen diseases, hematologic malignancies, and solid tumors are thought to be related to increases in the number of patients undergoing immunosuppressive therapy; these patients are more likely to receive penicillin and cephalosporin antibiotics. Consequently, increased *E. faecium* levels have been reported with changes in the gut microbiota of the large and small intestines [28].

Our present study identified male sex and hypoalbuminemia as independent risk factors of enterococcal infections. However, these are not specific to enterococcal infections. There have been several reports on the correlation between sex and infection prognosis. Angele et al. reported that men have poorer prognosis of septicemia than women, and the main reasons include its correlation with male sex steroids, X-chromosome mosaicism, etc. [29]. Hypoalbuminemia is also a known risk factor related to the onset of and mortality caused by various infections [30].

The mortality rates of enterococcal infections including VRE are high (25–50 %) since they often develop in compromised hosts [31–37]. Enterococci are not highly pathogenic per se, and their survival rates mainly depend on underlying disease severity. However, the mortality rates of enterococcal infections in the present study (8.2 %) were low compared to those of previous reports. The likely reasons for this are as follows: VRE was not detected in this study; anti-MRSA agents such as vancomycin were immediately administered against *E. faecium*, and treatment with appropriate antimicrobial agents was administered during the early stages of onset; and nearly all subjects were inpatients undergoing careful pathologic monitoring. Patients who contracted *E. faecium* infections from carriers had significantly longer hospital stays on univariate analysis. This is probably because of the increased condition severity of the patients with *E. faecium* infections as well as a high degree of drug resistance that necessitated long-term antimicrobial agent use [35–37].

## Conclusions

This study revealed the independent risk factors for *E. faecalis* and *faecium* infections. Significant risk factors for infection on multivariate analysis were structural abnormalities of the urinary tract, abdominal surgery, immunosuppressive agent use, in situ device use, male sex, and hypoalbuminemia. There was a significant correlation between liver transplantations and infections with *E. faecium* compared to *E. faecalis*. In cases complicated by bacteremia or intra-abdominal infections, enterococci are pathogenic microorganisms with a high mortality rate that must be handled carefully. Studies on risk factors for the onset of enterococcal infections provide useful information for clinicians, enabling improved prognosis, reduced hospitalization, and the proper use of antimicrobial agents through early treatment intervention. Nevertheless, additional prospective studies are necessary to confirm the present findings.

## Consent

Written informed consent was obtained from the patient for the publication of this report and any accompanying images.

## Abbreviations

*E. faecalis*: *Enterococcus faecalis*
*E. faecium*: *Enterococcus faecium*
SPSS: Statistical Package for the Social Sciences
SD: Standard deviation
OR: Odds ratio
CI: Confidence interval
